# Effects of Vitamin D Supplementation on C-peptide and 25-hydroxyvitamin D Concentrations at 3 and 6 Months

**DOI:** 10.1038/srep10411

**Published:** 2015-06-22

**Authors:** Paulette D. Chandler, Edward L. Giovannucci, Jamil B. Scott, Gary G. Bennett, Kimmie Ng, Andrew T. Chan, Bruce W. Hollis, Nader Rifai, Karen M. Emmons, Charles S. Fuchs, Bettina F. Drake

**Affiliations:** 1 Division of Preventive Medicine, Brigham and Women’s Hospital, Boston, MA; 2 Harvard Medical School, Boston, MA; 3 Departments of Nutrition and Epidemiology, Harvard School of Public Health, Boston, MA; 4 Channing Division of Network Medicine, Department of Medicine, Brigham and Women’s Hospital, Boston, MA; 5 Department of Epidemiology and Biostatistics, College of Human Medicine, Michigan State University, East Lansing, MI; 6 Department of Psychology and Neuroscience, Duke University, Durham, NC; 7 Department of Medical Oncology, Dana-Farber Cancer Institute, Boston, MA; 8 Division of Gastroenterology, Massachusetts General Hospital, Boston, MA; 9 Division of Pediatrics, Medical University of South Carolina, Charleston, SC; 10 Division of Laboratory Medicine, Children’s Hospital of Boston, MA; 11 Center for Community-Based Research, Dana-Farber Cancer Institute, Boston, MA; 12 Department of Surgery, Division of Public Health Sciences, Washington University School of Medicine, St. Louis, MO

## Abstract

The link between African-Americans’ disproportionate rates of diabetes, obesity and vitamin D deficiency may be marked by C-peptide as an indicator of insulin secretion. We hypothesize that vitamin D supplementation will increase C-peptide, a marker of insulin secretion. During 3 winters from 2007-2010, 328 healthy African-Americans (median age, 51 years) living in Boston, MA were randomized into a 4-arm, double-blind trial for 3 months of placebo, 1000, 2000, or 4000 IU of vitamin D3. The differences in non-fasting C-peptide between baseline and 3 months were −0.44 ng/mL for those receiving placebo, −0.10 ng/mL for those receiving 1000 IU/d, 0 ng/mL for those receiving 2000 IU/d, 1.24 ng/mL for those receiving 4000 IU/d (C-peptide increased 0.42 ng/mL for each additional 1000 IU/d of vitamin D3, p < 0.001). Vitamin D supplementation increased C-peptide in overweight African-Americans and may be compatible with other recommendations for diabetes prevention and management including weight loss and increased physical activity.

Dysregulation of insulin-mediated metabolic pathways has emerged as as an underlying mechanism through which vitamin D deficiency, diabetes and obesity may be linked. Vitamin D deficiency is associated with insulin resistance^1^,^2^ and pancreatic beta cell dysfunction. Vitamin D activity increases insulin release [phase 1] and insulin secretion [phase 2] from pancreatic islet beta cells (beta cells) *in vitro* and in vitamin D-deficient animals^3^. C-peptide hormone is an indicator of insulin secretion^4^. C-peptide, a connecting protein to insulin, is removed when insulin is released and secreted in the first and second phases of insulin secretion^5^.

Optimal vitamin D homeostasis may be essential for both insulin secretion and action, two fundamental features in the pathogenesis of insulin resistance and diabetes^6^. African-Americans experience a disproportionately high prevalence of vitamin D deficiency and increased risk for diabetes^7^. In this ancillary study of overweight and obese African-Americans, we hypothesize that vitamin D supplementation will increase non-fasting C-peptide, a marker of insulin secretion.

## Materials and Methods

This is a prospective, randomized, double-blind, placebo-controlled clinical trial of oral cholecalciferol (vitamin D3) in a community-based African-American population (ClinicalTrials.gov NCT00585637). The primary aim was to examine the effect of daily supplementation (placebo, 1000 international units (IU), 2000 IU and 4000 IU) of vitamin D3 on plasma 25(OH)D concentrations. A secondary aim was to evaluate the effect of daily vitamin D3 supplementation on C-peptide concentrations. This trial focused on African-Americans because African-Americans have higher rates of vitamin D deficiency compared to Whites^8^ or Africans^9^. Participants received daily oral supplementation during early winter (November or December) for 3 months (completed in February or March). All capsules contained 200 mg of calcium in the form of calcium carbonate. Patients were followed-up at 3 months and 6 months.

### Treatment

Participants were assigned to four arms consisting of placebo, 1,000 IU/day, 2,000 IU/day, or 4,000 IU/day of vitamin D3 for three months in a 1:1:1:1 ratio using block randomization stratified by age, sex, and enrollment month (Supplemental Figure 1). BMI was not included in matching randomization. Study statisticians generated the random allocation sequence and subjects were enrolled by research assistants. All capsules also contained 200 mg of calcium supplemented as calcium carbonate (Pharmavite LLC, Mission Hill, CA). Calcium was included because prior studies have shown that African-Americans have low calcium intake^10^. All capsules were indistinguishable, and both participants and research staff were blinded to treatment assignment. Study medications were started in early winter (November or December) and were taken orally once daily for 3 months (completed in February or March) calcium >2.62 mmol/L (10.5 mg/dL) was immediately discontinued from the study and the primary care physician was notified.).

### Measurement of 25(OH)D and C-peptide Concentrations

Plasma samples were collected in lavender-top evacuated tube which contained liquid EDTA at baseline, 3 and 6 months for 25(OH)D determination. Assays were performed in a single batch using a radioimmunoassay (DiaSorin Inc) in the laboratory of Bruce Hollis (Medical University of South Carolina, Charleston, SC). Masked quality-control samples were interspersed and all laboratory personnel blinded. The mean CV of the assay was 9%. Non-fasting C-peptide plasma samples were collected. In the lab of Nader Rifai (Boston Children’s Hospital, Boston, MA), C-peptide was measured by a competitive electrochemiluminescence immunoassay on the 2010 Elecsys autoanalyzer (Roche Diagnostics, Indianapolis, IN) with a coefficient of variation of 3%.

Non-fasting C-peptide correlates well with both fasting and glucagon-stimulated C-peptide, with correlation values of 0.78 and 0.77, respectively^11^. The primary endpoints of the study were changes in C-peptide from baseline to 3-month follow-up (post supplementation).

### Recruitment and Randomization

Participants in Open Doors to Health (ODH), a colorectal cancer (CRC) prevention intervention study in 12 public housing communities in the Boston metropolitan area and community organizations^12^, were invited to participate if they were 30 to 80 years old, understood written and spoken English, self-identified as Black^13^,^14^,^15^, and had permission from their primary care doctors. A total of 328 individuals were enrolled. Participants were recruited over 3 winters from 2007-2010. Exclusion criteria included pregnancy, renal disease, pre-existing parathyroid, thyroid, or calcium metabolism disorders, sarcoidosis, requirement for calcium channel blockers, type I diabetes, and active malignancies (other than non-melanoma skin cancer).

### Safety and Compliance

Participants were followed for toxicity and compliance every 2 weeks by phone and every 4 weeks in person during supplementation. To assess signs of elevated calcium, participants were educated on the potential symptoms of hypercalcemia and advised to contact study coordinators if symptoms occurred. At each adverse event assessment, study staff ascertained absence of symptoms (such as muscle aches, fatigue, excessive thirst, frequent urination, change in appetite, and changes to the skin [eg, pruritus], and nausea). In addition, serum calcium was measured in subjects who were taking hydrochlorothiazide (84 participants) at 4 to 6 weeks following study initiation and again at 12 weeks. An additional subset of control participants (44 participants), who did not take hydrochlorothiazide, also underwent calcium assays at 3 months. Plasma total calcium was analyzed using standard auto analyzer methodology. Any subject found to have serum calcium >10.5 mg/dL was immediately discontinued from the study and the primary care physician was notified. In addition, as part of the routine toxicity assessments, participants who reported any symptoms possibly associated with hypercalcemia had to undergo measurement of serum calcium at the time of the adverse event report. Electronic pill-dispenser systems and pill counts were also used to track compliance with study supplementation.

Participants were additionally asked to complete questionnaires at baseline, 3 and 6 months that addressed dietary and lifestyle behaviors, socioeconomic and demographic factors along with medication use. Further details of study procedures are presented elsewhere^16^. Information about diabetes medications or type 2 diabetes was not collected.

#### Statistical Analysis

Differences in the baseline characteristics of participants across the 4 treatment groups were compared using the Kruskal-Wallis test for continuous variables and a χ2 test for categorical comparisons. The primary end points were 3-month change in C-peptide at the end of treatment. For our primary analysis, we used linear regression with the dose of vitamin D3 (per 1000 IU/d) as the independent variable and the 3-month change in C-peptide as the dependent variable. For our secondary analysis, we stratified by baseline plasma 25(OH)D (<20 ng/mL, > 20 ng/mL) and used linear regression with the dose of vitamin D3 (per 1000 IU/d) as the independent variable and the 3-month change in C-peptide as the dependent variable. We also adjusted for baseline C-peptide values in the linear regression models to see whether vitamin D had more effect in those who had the least abnormal C-peptide concentrations. As an exploratory analysis, baseline C-peptide concentrations were grouped into tertiles to assess the effect of baseline C-peptide on C-peptide response to vitamin D supplementation. C-peptide response to vitamin D supplementation was examined in each treatment group after stratification into tertiles by baseline C-peptide values (Tertile 1 is lowest C-peptide concentration). The analyses were done by orginially assigned treatment groups.

#### Power

For C-peptide, a minimum sample size estimate of 84 subjects per arm was required to obtain 80% power to detect a 0.5 difference in the mean. Statistical power for this trial was based on 80 subjects per arm. Using a two-sided t-test at the 0.05 significance concentration, the minimum detectable difference in 25(OH)D between treatment arms was 5.3 and 6.2 ng/mL with 80% and 90% power, respectively. All statistical analyses were performed using SAS 9.2 (SAS Institute, Cary, NC). All participants provided written informed consent. The project was approved by the Institutional Review Boards of Harvard School of Public Health and Dana-Farber Cancer Institute. All procedures were followed in accordance with institutional guidelines.

## Results

### Baseline characteristics

Baseline characteristics for the participants are reported in Table 1. Approximately 50% of subjects were obese and about 75% were overweight. Among 328 participants, the median 25(OH)D concentration at baseline was 15.3 ng/ml and did not differ significantly between treatment arms (*P* *=* 0.63)(Table 1). The 3-month follow-up was completed in 288 participants (86%).

### Main analyses

For each additional 1000 IU/d of vitamin D3 taken, non-fasting C-peptide was significantly increased by 0.42 ng/mL (p <0.001) (Table 2). Vitamin D supplementation was stopped at 3 months.We examined the association of 3-month change in C-peptide with any dose of vitamin D3 supplementation (ie, all 3 treatment groups combined) compared with placebo and found that vitamin D3 supplementation at any dose significantly increased C-peptide by 0.82 ng/mL (p = 0.03). We examined the effect of change in plasma 25(OH)D with change in C-peptide. For each 1 ng/ml increase in 25(OH)D concentration between baseline and 3 months, there was a significant 0.02 ng/mL increase in C-peptide (p = 0.03). After adjusting for baseline C-peptide values, we examined the effect of change in plasma 25(OH)D with change in C-peptide. For each 1 ng/mL increase in 25(OH)D concentration between baseline and 3 months, there was a significant 0.02 ng/mL increase in C-peptide (p = 0.02).

We examined the change in C-peptide at 3 to 6 months. After no vitamin D supplementation for 3 months, the difference in C-peptide concentrations between 3 months and 6 months revealed no significant change in C-peptide per unit unit change in 25(OH)D (p = 0.17). (Table 2). From month 3 to month 6 with all 3 dose arms combined versus placebo, C-peptide decreased by −0.79 ng/mL (p = 0.02) in the period of no vitamin D supplementation. Per 1000 IU/d cessation of cholecalciferol supplementation, the 6 month C-peptide decreased by −0.30 ng/mL (p = 0.003). (Table 2).

### Stratified by Baseline C-peptide

C-peptide concentration increases varied with baseline C-peptide concentration. (Table 3) For treatment groups, placebo and vitamin D 1000 IU/d or 2000 IU/d, there was a significant negative correlation (correlation: −0.6 to −0.8, p < 0.0001) between baseline C-peptide concentrations and measured change in C-peptide concentration at 3 months in each dose group. For the vitamin D 4000 IU/d group no significant correlation between baseline C-peptide concentration and measured change in C-peptide concentration at 3 months (p = 0.34) was observed. At 3 months, the greatest change in C-peptide values occurred in the highest tertile of C-peptide concentrations; for Tertile 3, each additional 1000 IU/d of vitamin D3 corresponded to 0.70 ng/mL increase in C-peptide (p = 0.002).

### Stratified by Baseline 25(OH)D

We repeated our primary analysis after stratifying by baseline plasma 25(OH)D (<20 ng/mL, >20 ng /mL). The magnitude of association for each additional 1000 IU/d of vitamin D3 on C-peptide was greater among those whose baseline plasma 25(OH)D was > 20 ng/mL (0.60 ng/mL increase; p > 0.0001) than among those whose baseline plasma 25(OH)D was < 20 ng/mL (0.32 ng/mL increase; p = 0.02). However these effects were not statistically different from each other (P interaction = 0.20).

### Stratified by HCTZ

Of the 134 (41%) of participants using anti-hypertensives, 84 were on hydrochlorothiazide (HCTZ). No significant interaction between hydrochlorthiazide use and change in C-peptide with vitamin D supplementation was observed (p for interaction = 0.32). Plasma C-peptide increased in HCTZ and non-HCTZ users (HCTZ:0.42 ng/mL 95% CI [0.04,0.80]; p = 0.03; for each additional 1000 IU/d of vitamin D3; non-HCTZ: 0.42 ng/mL 95% CI [0.17,0.67];p = 0.001).

### Adverse Events

There were 5 isolated incidences of mild hypercalcemia which were in the reference range on repeated sampling^17^. Vitamin D supplements were discontinued in the 4 participants with mild hypercalcemia at 1 month. There were no episodes of nephrolithiasis^17^.

## Discussion

In this group of mostly overweight African-Americans, vitamin D3 supplementation increased C-peptide concentrations, but this effect was primarily observed for vitamin D3 4000 IU/d. C-peptide concentrations declined after discontinuation of the vitamin D supplementation as noted by the reduction in C-peptide at 6 months. We showed increases in non-fasting C peptide, a marker of insulin secretion, only with the largest doses, that were independent of age and degree of obesity. In stratified analyses by baseline C-peptide, C-peptide increases varied with baseline C-peptide. Vitamin D had the greatest effect on increase in C-peptide in those with the highest C-peptide levels. This dose-dependent effect of vitamin D on increasing C-peptide has been observed in a variety of settings including uremia^18^, healthy adults^19^, type 2 diabetes^20^, South Asians with dysglycaemia^21^, but has not previously been shown in African Americans. Boucher *et al.*^21^ demonstrated that the largest C-peptide responses after vitamin D supplementation with an oral glucose tolerance test (OGTT) were found in those whose baseline pre-supplementation C-peptide response at OGTT were the highest. Bouchard *et al.*^21^ suggested that vitamin D replacement may need to start before islet dysfucntion is marked if that dysfunction is to be corrected. Furthermore, a recent study has shown an increase in C-peptide with vitamin D supplementation in non-diabetic individuals^22^.

C-peptide hormone is an indicator of insulin secretion^4^. Since insulin secretion and insulin resistance are positively correlated in type 2 diabetes, at least in the early and middle stages of disease, C-peptide is positvely correlated with insulin resistance^23^,^24^. African-Americans tend to have poor vitamin D status^25^ and increased risk for type 2 diabetes. This may be clinically important because correction of chronic vitamin D deficiency may provide a safe and effective approach to reduce the risk of T2D and T2D complications and would be compatible with other clinical recommendations including weight loss and increased physical activity.

Prior literature has identified an association of insulin sensitivity in African-Americans with dietary vitamin D by using a robust measure of insulin sensitivity, the frequently-sampled intravenous glucose tolerance test (FSIGT) and a commonly used surrogate of whole body insulin resistance, Homeostasis Model Assessment of Insulin Resistance (HOMA-IR). Alvarez *et al.* reported that vitamin D intake was inversely associated with HOMA-IR and the relationship was independent of age, total body fat, and energy intake^26^. Furthermore, they found dietary vitamin D to be associated with insulin sensitivity in African-Americans but not European-Americans^26^. Similarly, Harris *et al.* documented a significant decrease in insulin sensitivity and an increase in C-peptide secretion and other measures of insulin secretion such as FSIGTS with no effects on glycemia in overweight or obese prediabetic African-Americans who received either 4000 IU vitamin D per day compared to those who receivedplacebo for 12 weeks^27^. In contrast, a small randomized control trial of Danish type 2 diabetes patients showed a borderline increase in C-peptide production with vitamin D supplementation (11,200 IU/d for 2 weeks followed by 5,600 IU/d for 10 weeks) versus placebo for 12 weeks without a change in other measures of insulin sensitivity such as HOMA-IR^28^.

Beta cell failure is key to the development and progression of T2D^29^. It antedates and predicts diabetes onset and progression^30^. This study sheds light on how vitamin D supplementation may impact the natural history of beta cell failure through C-peptide secretion, which is released in the first and second phases of rises in serum insulin concentrations following glucose challenge from the beta cells of the pancreas^31^. Its longer half-life, renal clearance, and equimolar release make C-peptide a good proxy for estimating insulin secretion^31^. In this study, higher baseline C-peptide concentrations were associated with greater increase in C-peptide after vitamin D supplementation. Since this study does not have measures of insulin resistance, we do not know if higher C-peptide levels are advantageous or deleterious. If there is beta-cell dysfunction, low C-peptide may reflect greater impairment of beta-cell function than high C-peptide. Furthermore, time since last meal may contribute to C-peptide concentrations, and we do not have the exact time since last meal to assess the influence of meal timing on C-peptide concentrations. C-peptide concentration may be higher because of recent meal^32^.

Since vitamin D receptor (VDR) as well as other parts of the vitamin D regulatory system are present at high concentrations in the pancreatic beta cell, vitamin D signaling may play a key role in the beta cell^3^. Vitamin D deficiency compromises *in vivo* and *in vitro* insulin secretion while vitamin D supplementation improves both^3^. In a recent meta-analysis on randomized control trials addressing the effect of vitamin D supplementation on glycemic outcomes and incident diabetes, a moderate but significant reduction in fasting glucose was found in the vitamin D treatment group compared with placebo. Furthermore, insulin resistance assessed by HOMA-IR or fasting insulin/C-peptide concentrations were also slightly decreased by vitamin D treatment, but there was no effect on C-peptide concentrations^33^. Another recent meta-analysis showed no effect of vitamin D supplementation on insulin resistance assessed by HOMA-IR or insulin secretion, but analysis included subjects with diabetes, prediabetes, and non-diabetics. Analysis was limited by heterogeneity of included trials^34^. Both analyses did not evaluate findings by race/ethnicity.

Our previous work showed that vitamin D supplementation reduced blood pressure^35^. Suppression of the local renin angiotensin (RAS) by calcitriol has been shown and may preserve pancreatic beta-cell function^36^. Thus, vitamin D may influence growth and differentiation of beta-cells^37^.

Strengths of this study include the double-blinded, randomized design in an understudied population, the use of multiple doses of vitamin D to determine the presence or absence of a threshold effect of vitamin D supplementation on C-peptide concentrations, the 3-month duration of the trial, the use of vitamin D supplementation in the winter months to limit confounding by sun exposure, and the measurement of C-peptide at multiple time points.

Limitations of our study include the lack of measurement of dysglycemia, insulin resistance, or sensitivity. Inability to determine if dysglycemia varied between groups and lack of measurment of peripheral response to insulin make it difficulty to interpret the results as having a positive impact on glycemic control. The groups are not large enough for the population prevalence of various degrees of C-peptide reduction due to islet dysfunction to be matched with any certainty. Although the comparable baseline mean C-peptides in the 4 groups are reassuring, we do not have other data to support this assumption of comparable islet dysfunction. The availability of only non-fasting bloods added variability to the C-peptide measuremnt, though this should be equalized across groups due to randomization.Although indicators of glycemic status were not available, the study population likely included a substantial number of individuals with prediabetes or type 2 diabetes^38^. About 20% of obese African-Americans have diagnosed diabetes^17^ and about 30% of Americans have either undiagnosed diabetes or prediabetes^39^. The clinical significance of higher C-peptide levels is unknown in this population. Since our highest dose was 4000 IU/d, we were not able to evaluate the influence of higher vitamin D doses on plasma 25(OH)D. Other than hydrochlorothiazide, we had limited data on specific classes of anti-hypertensive agents used by participants.

In conclusion, vitamin D deficiency may increase risk for type 2 diabetes. The data from the present study show that vitamin D increases insulin secretion in African-Americans as in other ethnic groups. Larger and longer intervention studies are needed to determine fully the long term effects of vitamin D repletion on C-peptide secretion and glucose homeostasis in this group.

## Additional Information

**How to cite this article**: Chandler, P. D. *et al.* Effects of Vitamin D Supplementation on C-peptide and 25-hydroxyvitamin D Concentrations at 3 and 6 Months. *Sci. Rep.* **5**, 10411; doi: 10.1038/srep10411 (2015).

## Supplementary Material

Supplementary Information

## Figures and Tables

**Table 1:** Baseline characteristics.

CHARACTERISTIC*	VITAMIN D_3_ DOSE ASSIGNMENT (IU)			
	PLACEBO (n = 81)	1,000 (n = 81)	2,000 (n = 83)	4,000 (n = 83)
**Age (y)**	50.7 (44.1-58.0)^a^	51.1 (43.4-60.1)	50.3 (43.5-58.3)	51.3 (44.1-59.7)
**Sex, No. (%)**				
Male	27 (33.3)	22 (27.2)	28 (33.7)	29 (34.9)
Female	54 (66.7)	59 (72.8)	55 (66.3)	54 (65.1)
**BMI (kg/m**^**2**^)	31.2 (26.5-35.9)	30.5 (27.0-37.5)	31.9 (26.2-36.9)	31.4 (27.4-35.7)
**Biomarkers**				
C-peptide (ng/mL)	3.61 (2.73-5.67)	3.52 (2.46-5.80)	3.64 (2.51-5.35)	3.90 (2.75-6.11)
25 (OHD) (ng/mL)	15.1 (10.4-23.6)	16.2 (11.0-22.7)	13.9 (9.5-22.3)	15.7 (11.0-23.3)
**Smoking status, No. (%)**				
Never	33 (40.7)	36 (44.4)	33 (39.8)	44 (53.0)
Past	20 (24.7)	16 (19.8)	27 (32.5)	20 (24.1)
Current	28 (34.6)	29 (35.8)	23 (27.7)	19 (22.9)
**Frequency of exercise, (d/wk)**^b^	3.0 (0.5-5.0)	3.0 (1.0-5.0)	3.0 (0-5.0)	3.0 (0-5.0)
**Dietary vitamin D intake (IU)**^c^	147.3 (71.4-262.8)	162.5 (92.6-295.5)	144.0 (58.0-265.1)	198.1(83.2-306.4)
**Dietary calcium intake (mg)**^c^	277.0 (171.7-632.3)	422.9 (226.1-795.9)	318.8 (172.7-637.4)	445.9 (198.6-780.4)
**Regular multivitamin use,**^d^ **No. (%)**	10 (12)	18 (22)	15 (18)	22 (27)
**Regular vitamin D supplement use,**^d^ **No. (%)**	8 (10)	6 (8)	2 (2)	8 (10)
**Post-menopausal hormone use,**^e^ **No. (%)**	0	0	0	1 (0.5)
**Regular calcium supplement use,**^d^ **No. (%)**	7 (8.7)	9 (11.1)	7 (8.4)	9(10.8)
**History of cancer,**^f^ **No. (%)**	6 (7.4)	6 (7.4)	0	3 (3.6)
**History of hypertension, No. (%)**	35 (43.2)	35 (43.2)	36 (43.3)	35 (42.1)

^*^There were no significant differences in subject characteristics across supplementation arms except as noted for cancer.

^a^Median; 25^th^, 75^th^ percentiles in parentheses (all such values).

^b^Exercise defined as moderate to vigorous physical activity for at leaast 30 min, resulting in a faster-than-normal heart rate, sweating, and deep breathing.

^c^Refers to the intake during the preceding month.

^d^Defined as supplement use for 7 d/wk during the preceding month.

^e^Percentages calcuated from a total of 222 females.

^f^Reported cancers include breast cancer, cervical cancer, uterine cancer, lung cancer, prostate cancer, and sarcoma; p = 0.03.

**Table 2:** Effect of vitamin D supplementation on C-peptide (ng/mL) at 3 months and 6 months.

	Vitamin D Dose, IU/d					
**Parameter**	Placebo	1000	2000	4000	Mo Change in C-peptide or 25 (OH)D per 1000 IU/d#	P-Value
**n (at baseline)**	81	81	83	83		
Baseline C-Peptide*	4.46 (0.31)	4.37 (0.30)	4.16 (0.27)	4.49 (0.26)		
3 mo C-Peptide	3.83 (0.21)	4.31 (0.29)	4.19 (0.25)	5.72 (0.44)		
6 mo C-peptide	4.74 (0.30)	4.07 (0.30)	4.98 (0.31)	4.89 (0.49)		
Difference C-Peptide (0-3)	−0.44 (0.30)	−0.10 (0.29)	0 (0.28)	1.24 (0.38)	0.42 (0.11)	<0.0001
Difference C-peptide (3-6)	0.75 (0.27)	−0.27 (0.33)	0.86 (0.27)	−0.74 (0.31)	−0.30 (0.10)	0.003
Difference C-peptide (0-6)	0.20 (2.59)	−0.35 (2.76)	0.88 (2.83)	0.37 (3.71)	0.10 (0.12)	0.38
**Baseline 25(OH)D**	17.07 (1.03)	17.33 (1.00)	16.12 (0.98)	17.64 (0.98)		
3 mo 25(OH)D	14.23 (0.96)	28.12 (1.12)	35.48 (1.21)	47.07 (1.22)		
6 mo 25(OH)D	19.07 (0.99)	22.26 (0.96)	26.77 (1.02)	31.46 (0.81)		
Difference 25(OH)D (0-3)	−2.58 (0.66)	11.01 (1.22)	19.21 (1.21)	29.71 (1.30)	7.73 (0.39)	<0.0001
Difference 25(OH)D (3-6)	4.79 (0.77)	−5.80 (1.10)	−9.20 (0.91)	−15.72 (0.93)	−4.74 (0.32)	<0.0001
Difference 25(OH)D (0-6)	1.83 (0.86)	5.02 (0.87)	10.29 (0.95)	13.82 (0.99)	3.04 (0.31)	<0.001

^#^0-3 month change in C-peptide per 1000 IU/d of vitamin D supplementation, 3-6 month change in C-peptide per 1000 IU/d of vitamin D supplementation, 0-6 month change in C-peptide per 1000 IU/d of vitamin D supplementation; 0-3 month change in 25(OH)D per 1000 IU/d of vitamin D supplementation, 3-6 month change in 25(OH)D per 1000 IU/d of vitamin D supplementation, 0-6 month change in 25(OH)D per 1000 IU/d of vitamin D supplementation.

^*^C-peptide ng/mL: Mean(Standard Error) IU, international unit.

Difference C-Peptide (0-3)= Month 3 C-peptide concentration ng/ml- Month 0 C-peptide concentration ng/ml: Mean (SE). Difference C-Peptide (3-6)= Month 6 C-peptide concentration ng/mL- Month 3 C-peptide concentration ng/mL: Mean (SE). Difference C-Peptide (0-6)= Month 6 C-peptide concentration ng/mL- Month 0 C-peptide concentration ng/mL: Mean (SE). The numbers do not always sum to group totals due to missing information for some variables.

**Table 3:** Effect of vitamin D supplementation on C-peptide (ng/mL) at 3 months and 6 months stratified by baseline C-peptide.

	Vitamin D Dose, IU/d					
Parameter	Placebo	1000	2000	4000	Mo Change in C-peptide per 1000 IU/d#	P-Value
No. (at baseline)	81	81	83	83		
C-Peptide, Tertile 1*						
**No. in Tertile**	27	30	28	25		
Baseline	2.10 (0.14)	2.06 (0.13)	1.97 (0.14)	2.13 (0.14)		
3 mo C-Peptide	2.85 (0.25)	3.28 (0.33)	3.38 (0.37)	3.91 (0.55)		
6 mo C-peptide	3.78 (0.40)	2.67 (0.34)	4.67 (0.59)	2.99 (0.47)		
Difference C-Peptide (0-3)	0.76(0.21)	1.24 (0.24)	1.41 (0.30)	1.78 (0.53)	0.24 (0.12)	0.04
Difference C-peptide (3-6)	1.66 (0.33)	0.63 (0.27)	2.69 (0.52)	0.85 (0.53)	−0.31 (0.17)	0.07
Difference C-peptide (0-6)	0.95 (0.41)	−0.62(0.27)	1.14 (0.62)	−0.83 (0.57)	−0.08 (0.15)	0.57
C-Peptide, Tertile 2*						
**No. in Tertile**	26	24	29	30		
Baseline	3.79 (0.12)	3.76 (0.12)	3.85 (0.12)	3.91 (0.11)		
3 mo C-Peptide	4.08 (0.42)	4.20 (0.35)	4.04 (0.39)	5.50 (0.54)		
6 mo C-peptide	4.47 (0.39)	4.37 (0.50)	4.58 (0.42)	4.37 (0.48)		
Difference C-Peptide (0-3)	0.22 (0.46)	0.35 (0.37)	0.20(0.38)	1.55(0.57)	0.34 (0.16)	0.03
Difference C-peptide (3-6)	0.59 (0.40)	0.52 (0.47)	0.74 (0.38)	0.38 (0.53)	−0.34 (0.16)	0.03
Difference C-peptide (0-6)	0.49 (0.38)	0.17 (0.62)	0.67 (0.32)	−0.90 (0.51)	−0.05 (0.15)	0.74
C-Peptide, Tertile 3*						
**No. in Tertile**	28	27	26	28		
Baseline	7.36 (0.54)	7.50 (0.40)	6.87 (0.44)	7.22 (0.33)		
3 mo C-Peptide	4.68 (0.34)	5.63 (0.63)	5.20 (0.48)	7.68 (0.98)		
6 mo C-peptide	5.89 (0.60)	5.42 (0.55)	5.86 (0.58)	7.20 (1.16)		
Difference C-Peptide (0-3)	−2.33 (0.58)	−2.06 (0.59)	−1.71 (0.54)	0.39 (0.84)	0.70 (0.22)	0.002
Difference C-peptide (3-6)	−1.53 (0.55)	−2.17 (0.74)	−1.10 (0.59)	−0.09 (1.06)	−0.24 (0.20)	0.23
Difference C-peptide (0-6)	0.75 (0.55)	−0.24 (0.80)	0.79 (0.46)	−0.48 (0.56)	0.43 (0.25)	0.09

^#^0-3 month change in C-peptide per 1000 IU/d of vitamin D supplementation, 3-6 month change in C-peptide per 1000 IU/d of vitamin D supplementation, 0-6 month change in C-peptide per 1000 IU/d of vitamin D supplementation adjusted for baseline C-peptide.

^*^C-peptide ng/mL: Mean(Standard Error) IU, international unit.

Difference C-Peptide (0-3)= Month 3 C-peptide concentration ng/ml- Month 0 C-peptide concentration ng/ml: Mean (SE). Difference C-Peptide (3-6)= Month 6 C-peptide concentration ng/mL- Month 3 C-peptide concentration ng/mL: Mean (SE). Difference C-Peptide (0-6)= Month 6 C-peptide concentration ng/mL- Month 0 C-peptide concentration ng/mL: Mean (SE). The numbers do not always sum to group totals due to missing information for some variables.
