# Supplementary material for: Effects of Vitamin D Supplementation on C-peptide and 25-hydroxyvitamin D Concentrations at 3 and 6 Months
Source: Sci Rep. 2015 Jun 22;5:10411. doi: 10.1038/srep10411 (PMC4476090; doi:10.1038/srep10411)

Effects of Vitamin D Supplementation on C-peptide and 25-hydroxyvitamin D Concentrations at 3 and 6 Months

Authors and Affiliations:
Paulette D. Chandler; Edward L. Giovannucci; Jamil B. Scott; Gary G. Bennett; Kimmie Ng;[2]Andrew T. Chan; Bruce W. Hollis; Nader Rifai; Karen M. Emmons; Charles S. Fuchs; Bettina F. Drake

Supplemental Figure 1. Consort Diagram

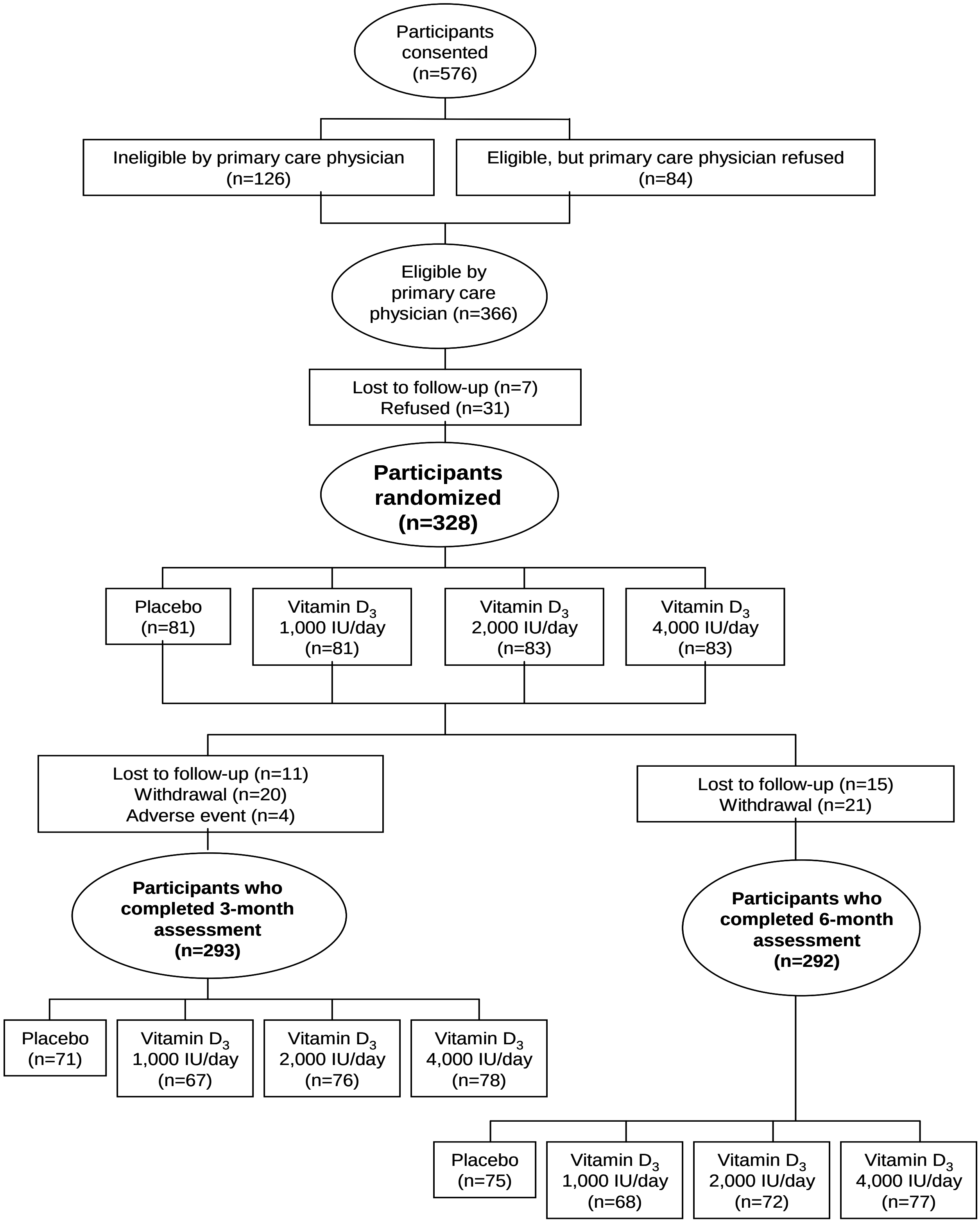

Supplement: Supplementary Information [file srep10411-s1.pdf]
